# Worsening versus advanced heart failure: Management and challenges

**DOI:** 10.1002/ehf2.15437

**Published:** 2025-10-04

**Authors:** Alberto Palazzuoli, Marco Giuseppe Del Buono, Giulia La Vecchia, Stephen J. Greene, Andrew P. Ambrosy, Ovidiu Chioncel, Finn Gustafsson, Selim R. Krim, Carl J. Lavie, Marianna Adamo, Tuvia Ben Gal, Oliviana Geavlete, Laura Antohi, Giuseppe Rosano, Sean Collins, Filippo Crea

**Affiliations:** ^1^ Cardiovascular Diseases Unit, Cardio‐Thoracic and Vascular Department Le Scotte Hospital, University of Siena Siena Italy; ^2^ Department of Cardiovascular Sciences Fondazione Policlinico Universitario A. Gemelli IRCCS Rome Italy; ^3^ Department of Cardiovascular and Pulmonary Sciences Catholic University of the Sacred Heart Rome Italy; ^4^ Centre of Excellence in Cardiovascular Sciences Isola Tiberina Hospital, Gemelli Isola Rome Italy; ^5^ Duke Clinical Research Institute Durham North Carolina USA; ^6^ Division of Cardiology Duke University School of Medicine Durham North Carolina USA; ^7^ Department of Cardiology Kaiser Permanente San Francisco Medical Center San Francisco CA USA; ^8^ Division of Research Kaiser Permanente Northern California Pleasanton California USA; ^9^ Institute of Cardiovascular Diseases ‘Prof. CC Iliescu’ University of Medicine Bucharest Romania; ^10^ Department of Cardiology Rigshospitalet, University of Copenhagen Copenhagen Denmark; ^11^ Section of Cardiomyopathy & Heart Transplantation, John Ochsner Heart and Vascular Institute Ochsner Clinic Foundation New Orleans Louisiana USA; ^12^ John Ochsner Heart and Vascular Institute Ochsner Clinical School‐The University of Queensland School of Medicine New Orleans Louisiana USA; ^13^ Department of Cardiology, Civil Hospital of Brescia University of Brescia Brescia Italy; ^14^ Heart Failure Unit, Cardiology Department, Rabin Medical Center, Faculty of Medicine Tel Aviv University Tel Aviv Israel; ^15^ Department of Human Sciences and Promotion of Quality of Life San Raffaele Open University of Rome Rome Italy; ^16^ Clinical Academic Group, Molecular and Clinical Sciences Research Institute City St George's, University of London London UK; ^17^ Department of Emergency Medicine and Veterans Affairs Tennessee Valley Healthcare System Geriatric Research, Education and Clinical Center (GRECC) Nashville Tennessee USA

**Keywords:** advanced heart failure, hospitalization, management, outcome, worsening heart failure

## Abstract

Heart failure (HF) is a progressive condition marked by recurrent episodes of symptom exacerbation, leading to worsening cardiac function, increased hospitalization and mortality risk. Worsening HF (WHF) and advanced HF (AdvHF) represent two distinct stages in this progression, each with unique clinical features and therapeutic needs. WHF is characterized by a deterioration of pre‐existing symptoms requiring intensified treatment, such as diuretic escalation, which often reflects disease progression. Conversely, AdvHF involves severe cardiac dysfunction with persistent symptoms despite optimal medical management, requiring advanced interventions such as inotropic support or heart transplant. Although both stages share some pathophysiological and clinical features, they differ significantly in haemodynamic profiles, disease severity and response to treatment. This review argues that recognizing the transition from WHF to AdvHF is a pivotal issue in patient care. We explore the distinct natural histories, clinical presentations and diagnostic markers of WHF and AdvHF to provide a framework for earlier, more targeted interventions aimed at altering the disease trajectory and preventing the decline associated with the advanced stage. While WHF symptoms are typically reversible with appropriate interventions, AdvHF represents the end stage of HF with often irreversible dysfunction and multi‐organ involvement. A clearer understanding and standardized definition of these phenotypes are essential for improving patient outcomes and guiding future clinical research.

## Graphical abstract

Clinical feature, circulating biomarkers and therapeutic management of patients with WHF versus AdvHF. *Abbreviations*: AdvHF, advanced heart failure; HF, heart failure; HTx, heart transplant; GDMT, guideline‐directed medical therapy; MCS, mechanical circulatory support; NYHA, New York Heart Association; WHF, worsening heart failure.

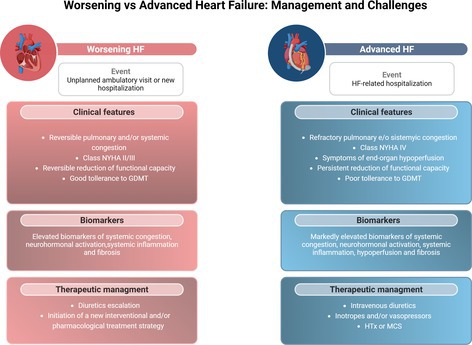

## Introduction

The clinical course of a patient with chronic heart failure (HF) is characterized by episodes of symptom exacerbation that lead to worsening cardiac function and an increased risk of hospitalization and mortality. Historically, these periods of deterioration have often been viewed monolithically. However, a crucial evolution in our understanding now distinguishes between two entities: worsening HF (WHF) and advanced HF (AdvHF), each with specific risk profiles, clinical presentation and severities. WHF is commonly understood as a crucial stage—an episode of worsening signs and symptoms that requires an urgent intensification of therapy, such as escalating diuretics. It is a high‐risk state, but one that is often reversible, presenting a crucial window of opportunity to restore stability and treatment optimization.^1^, ^2^, ^3^ In contrast, AdvHF represents a more severe impairment of cardiac function and an advanced stage of HF evolution, with patients experiencing persistent severe symptoms despite optimal medical management. These patients frequently require inotropic support, assessment for mechanical circulatory support (MCS) or heart transplant (HTx) and palliative care for those ineligible for these therapies.^4^, ^5^, ^6^, ^7^ While AdvHF and WHF share some clinical and pathophysiological similarities, they differ significantly in the haemodynamic profile, disease severity and progression.^8^, ^9^ This distinction is more than semantic; it represents a fundamental paradigm shift in how we approach diagnosis, risk stratification and treatment. The 2021 European Society of Cardiology (ESC) guidelines discuss AdvHF,^10^ while a 2023 ESC consensus paper formally introduced the concept of WHF,^1^ highlighting the recent efforts to refine this classification. However, a significant clinical challenge remains: A relevant overlap exists between these two entities, and a thorough comparative analysis of their similarities and differences is still lacking. This evolving classification underscores the need for a clearer framework to better identify and treat patients with progressive HF deterioration.^2^, ^11^ This review aims to bridge that gap by providing a comparative analysis of WHF and AdvHF. A clear distinction between these phenotypes is essential for tailoring management, allocating healthcare resources effectively and, ultimately, improving patient prognosis by intervening before the onset of irreversible end‐stage disease.

## Natural history of HF

**Figure 1:**
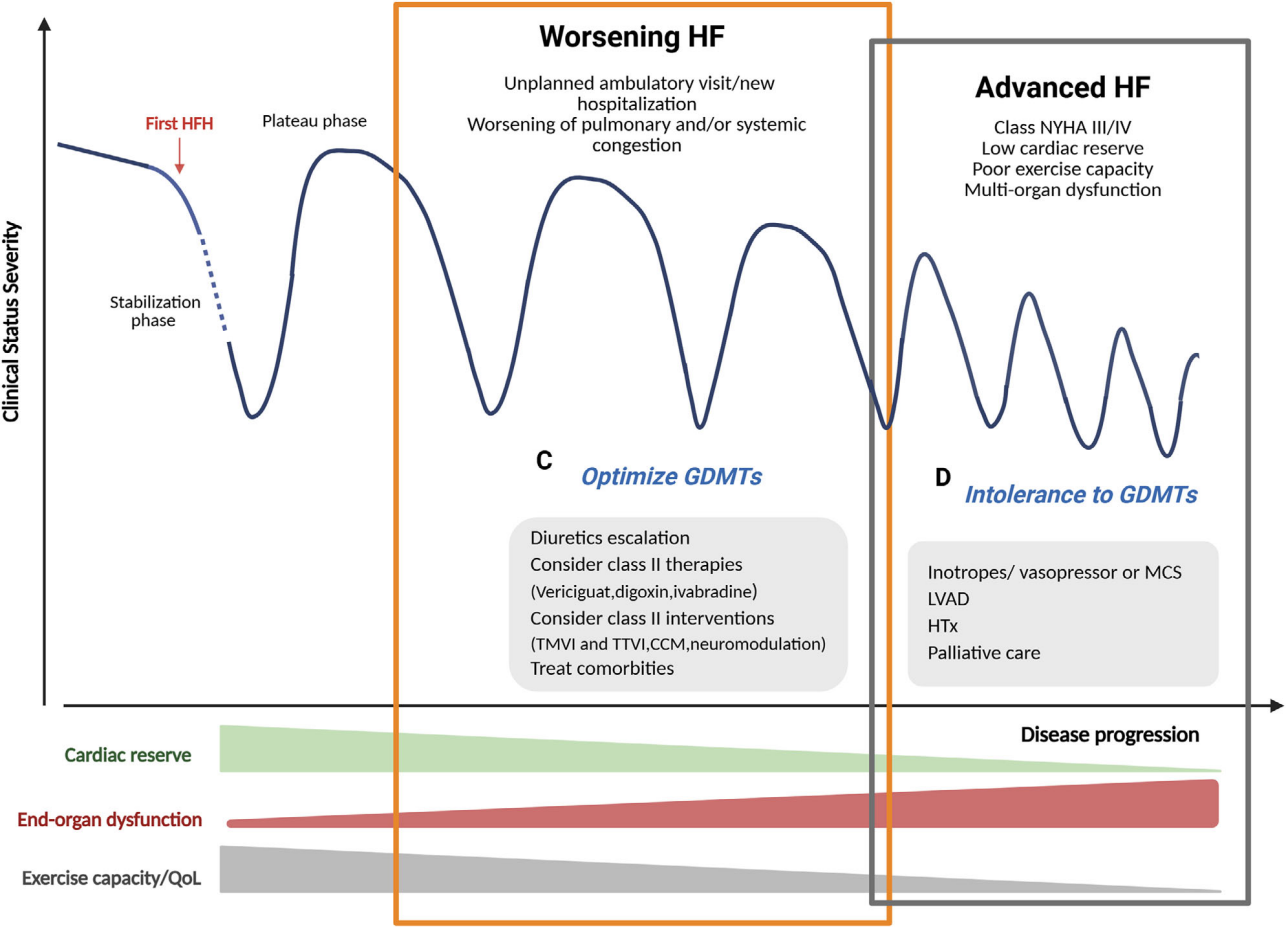
Different temporal trend clinical courses and clinical profiles associated with advanced versus worsening HF. *Abbreviations:* HF, heart failure; HFH, heart failure hospitalization; MCS, mechanical circulatory support; NYHA, New York Heart Association.

Throughout the clinical course of a patient with chronic, pre‐existing HF, WHF and AdvHF represent two distinct entities, each with specific risk profiles and severities. Traditionally, the natural course of HF follows a progressive trajectory marked by phases of stability, vulnerability and decline (*Figure* *1*). An initial vulnerable phase usually follows the first HF hospitalization (HFH), a critical period lasting at least 30 and 60 days post‐discharge but potentially extending up to 6 months.^8^, ^12^ This phase is associated with a markedly elevated risk of death or readmission, as patients remain susceptible to further decompensations despite guideline‐directed medical therapy (GDMT).^12^, ^13^ Following this high‐risk window is a plateau phase of relative symptomatic stability with variable duration, lasting anywhere from 6 months to 5 years, depending on individual disease progression and response to treatment.^13^, ^14^ During this period, patients may experience minimal symptom fluctuations, often maintaining functional capacity with appropriate medical and lifestyle interventions. However, underlying pathophysiological processes, including progressive myocardial remodelling, neurohormonal activation and end‐organ dysfunction, continue to evolve, setting the stage for future deterioration. This stability may be interrupted by episodes of WHF, where symptoms worsen either gradually or abruptly, necessitating recurrent HFHs or urgent medical visits.^15^, ^16^ In the context of WHF, recurrent decompensations may arise even during seemingly stable periods, often due to transient haemodynamic imbalances or non‐cardiac stressors. Current instability, although reversible with appropriate interventions, reflects a progressive disease trajectory requiring intensified monitoring and therapy adjustments. AdvHF represents the terminal phase of HF, where patients experience a profound and irreversible decline in cardiac function, marked by persistent symptoms despite optimal GDMT. Unlike WHF, AdvHF is characterized by a further loss of cardiac reserve, permanent exercise intolerance and systemic multi‐organ dysfunction. Patients in this stage frequently develop refractory congestion, cachexia and renal or hepatic impairment.^17^ The prevalence of AdvHF tends to increase in the general HF population due to the ageing of the population, improved therapeutic options and better survival after acute coronary attack. Therefore, the availability of recent evidence‐based therapies acting in all HF subtypes contributes to the relevant life prolongation. However, there are geographical variations in AdvHF epidemiology with a substantial lack of data from developing countries, where HF exhibits different features and courses compared with those observed in Western countries. Although WHF and AdvHF share common pathophysiological mechanisms, their clinical implications differ significantly for precipitating factors, patient history, cardiac status and haemodynamic status. Understanding these distinct trajectories is crucial for optimizing treatment strategies, identifying appropriate candidates for advanced therapies and ultimately improving patient outcomes.

## Definitions

### Worsening HF

WHF is clinically defined by the Heart Failure Association of the ESC as the worsening symptoms and signs of HF in patients with a pre‐existing diagnosis, necessitating treatment intensification.^1^ A similar definition from a US review perspective specifically highlights the worsening of HF signs and symptoms following a period of clinical stability.^3^, ^9^ While patients hospitalized for HF face a significantly higher risk of subsequent clinical events compared to non‐hospitalized patients, WHF episodes can occur in various settings, including during hospitalization, an emergency department visit or an unscheduled ambulatory visit.^13^, ^17^ Operationally, WHF is primarily identified by the need to intensify therapies, such as administering intravenous (IV) diuretics, escalating oral diuretic doses or requiring unplanned clinic visits. However, because the definition is limited by heavy reliance on subjective symptoms rather than well‐defined diagnostic criteria and objective haemodynamic cut‐off, achieving a standardized definition remains challenging.^13^, ^17^

A further distinction in terminology has been proposed between WHF and decompensated HF (DHF). WHF is a broader concept, representing a trajectory of deterioration in a patient's chronic HF condition over time. DHF is viewed as a more specific pattern within the WHF trajectory. In DHF, therapies beyond standard GDMT are required to stabilize the patient. Thus, while all patients with DHF are experiencing WHF, not all individuals with WHF have reached the critical point of decompensation necessitating such escalated interventions. This differentiation is crucial for refining patient characterization, guiding therapeutic strategies and informing the design of clinical trials.^18^

Residual congestion following discharge, whether clinically evident or subclinical, remains a significant factor in the recurrence of WHF events. Furthermore, various precipitating factors can destabilize HF status, potentially leading to hospitalization. These triggers include patient non‐adherence to dietary advice (e.g., salt restriction) or prescribed medications, as well as a range of comorbid conditions. Common triggers involve atrial arrhythmias (e.g. atrial fibrillation), valvular heart disease and non‐cardiovascular disorders such as renal disease, sleep‐disordered breathing, iron deficiency, thyroid disorders and exacerbations of chronic obstructive pulmonary disease. Additionally, the chronic use of certain medications—including non‐steroidal anti‐inflammatory drugs (NSAIDs), corticosteroids, immunomodulators, monoclonal antibodies and traditional anti‐cancer treatments—can act as precipitating factors that accelerate the natural progression of HF. Collectively, all these factors can contribute to WHF recurrence and frequently necessitate specific management strategies targeted at the trigger. While many factors can precipitate a worsening of HF signs and symptoms, certain situations should be carefully considered and potentially distinguished from a true WHF event that signifies a progression of the underlying disease process despite stable therapy. This includes instances where there is a gross lack of adherence to medical therapy, which may indicate undertreated chronic HF rather than a worsening despite stable therapy. Similarly, decompensation clearly and predominantly caused by acute secondary diseases, such as acute coronary syndrome or severe acute respiratory infections, may also warrant differentiation.^9^, ^14^ Although the consensus statement by the Heart Failure Association of the ESC considers some of these factors as exclusion criteria for WHF, it is often challenging to discern the primary pathophysiological mechanisms involved. Rather than representing discrete causes, these precipitating factors may reflect a broader vulnerability of the underlying substrate. Consequently, their consistent presence should not be used as grounds for exclusion, but rather as indicators that warrant a comprehensive and individualized management approach.^2^, ^15^, ^19^

### Advanced HF

According to the most recent ESC guidelines, AdvHF includes a range of structural and functional heart diseases that result in refractory or persistent HF symptoms (NYHA Class III or IV) despite optimal medical and device therapy.^4^, ^6^, ^19^ This definition emphasizes the persistence of subjective symptoms rather than relying solely on the degree of cardiac dysfunction. Although a severely reduced left ventricular (LV) ejection fraction (LVEF < 30%) is often observed, AdvHF also encompasses patients with HF with preserved ejection fraction, non‐operable advanced valvular pathologies, severe non‐operable congenital abnormalities or significant right ventricular (RV) dysfunction.^7^, ^20^ Additional criteria for AdvHF include (1) acute clinical events, defined as episodes of pulmonary or systemic congestion requiring high‐dose IV diuretics (or diuretic combinations), low cardiac output necessitating inotropes or vasoactive drugs, or malignant arrhythmias leading to more than one unplanned visit or hospitalization in the last 12 months; (2) decline in functional capacity, defined as a significant reduction in exercise capacity, as evidenced by a 6‐min walk test distance of less than 300 m and a peak oxygen consumption (VO_2_) of less than 12 mL/kg/min (or less than 14 mL/kg/min in patients intolerant to beta‐blockers) or below 50% of the predicted value.^21^, ^22^

The INTERMACS (Interagency Registry for Mechanically Assisted Circulatory Support) has further stratified AdvHF into seven profiles. These range from patients with limited exercise capacity (Profiles 7–5) to those requiring inotropic or mechanical support due to organ hypoperfusion, systemic vasoconstriction and persistent congestion (Profiles 3–1).^23^ This broad spectrum of clinical presentations may pose challenges for physicians, particularly when differentiating between the less severe phenotypes of AdvHF.

Definitions and clinical features of WHF versus AdvHF are summarized in *Table* *1*.

**Table 1:** Main differences and characteristics of worsening and advanced heart failure.

Characteristic	Worsening heart failure	Advanced heart failure
Definition	Worsening symptoms and signs in patients with pre‐existing HF despite background HF therapy, requiring urgent therapy (e.g., hospitalization, outpatient IV diuretic administration, escalation of oral diuretics)	Persistent or refractory HF symptoms (NYHA Class III or IV) and/or persistent end‐organ dysfunction, despite optimal therapy
Key features	Episodes occurring after clinical stability; primarily defined by therapy intensification (IV/oral diuretics, ambulatory visits)	Structural/functional heart disease causing persistent severe symptoms and/or end‐organ dysfunction
Triggers	Antecedent precipitating events such as arrhythmia, infection, other comorbidities, or treatment disruptions possibly present	Pump failure, exhaustion of compensatory mechanisms, multiple systemic organ dysfunction
Clinical events	Possible occurrence of WHF episodes regardless of care settings; residual congestion increasing recurrence risk	Frequent hospitalizations; need for high‐dose IV diuretics, inotropes or vasoactive drugs
Functional decline	Based on subjective symptoms rather than haemodynamic measures	Reduced exercise capacity (6MWT < 300 m, VO_2_ < 12–14 mL/kg/min)
Prognosis	Variable; worse prognosis in patients with recurrent episodes and those requiring multiple hospitalizations	Poor prognosis; typically associated with high morbidity and mortality rates despite treatment

Abbreviations: HF, heart failure; IV, intravenous; NYHA, New York Heart Association; VO_2_, peak oxygen consumption; RV, right ventricular; 6MWT, 6‐min walk test; WHF, worsening heart failure.

## Distinguishing the phenotypes

### Clinical presentation

WHF presents with an exacerbation of previously stable symptoms, which is prone to recurrent HFH, with a mortality rate at 1 year of around 20%.^2^, ^24^ Although clinical presentation can differ depending on precipitating factors, comorbidities and speed of deterioration, peripheral and pulmonary congestion due to fluid retention and redistribution are common, usually without signs of organ hypoperfusion. The arrhythmic burden may consist of new‐onset atrial fibrillation and increased ectopic ventricular arrhythmias.^25^

**Figure 2:**
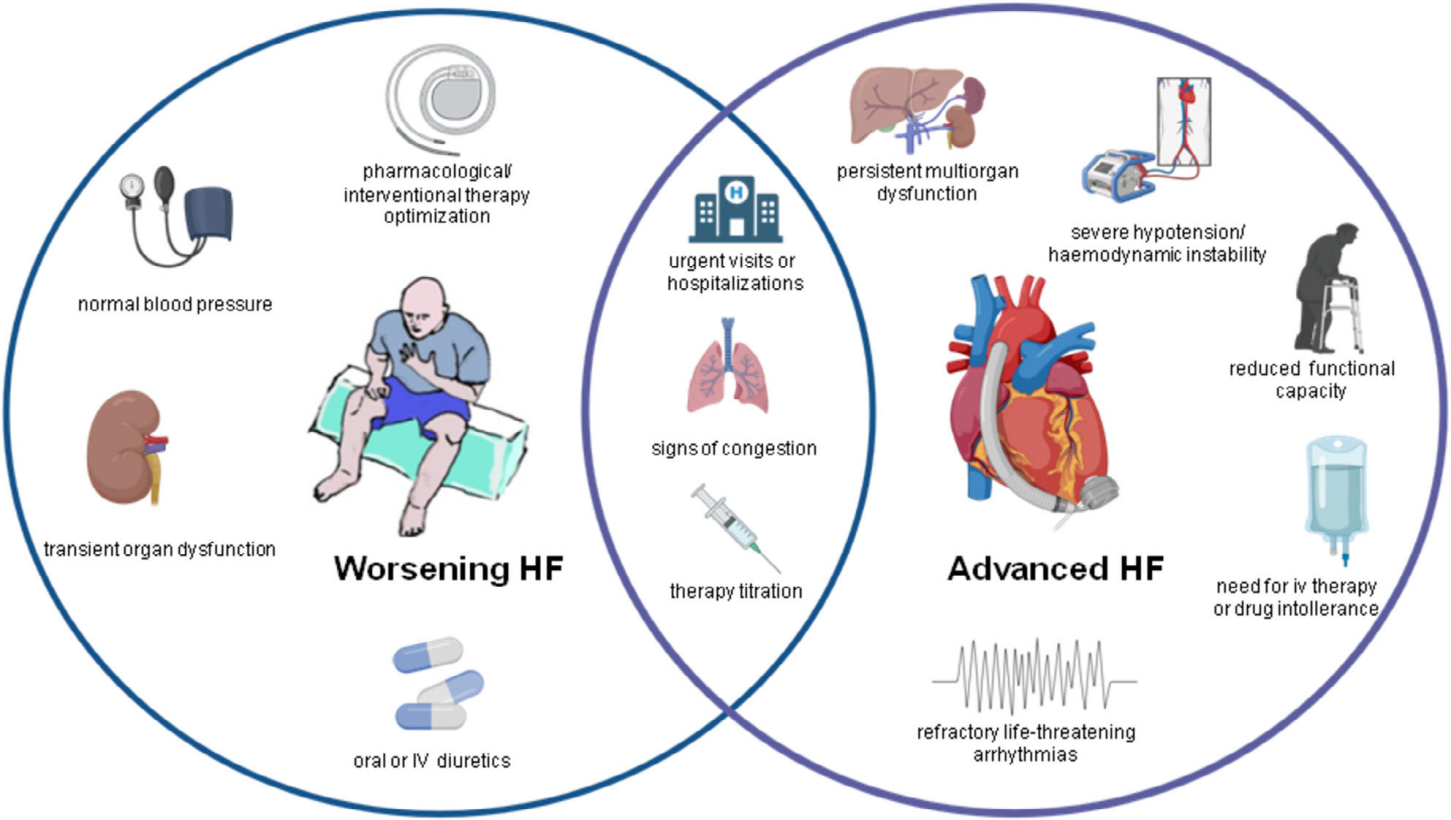
Despite apparently similar clinical presentation, advanced HF may be distinguished from worsening HF by a specific haemodynamic pattern and presentation. *Abbreviations*: HF, heart failure; IV, intravenous.

Conversely, AdvHF is predominantly characterized by cardiac and haemodynamic deterioration with significant limitations in physical activity such as dyspnoea at rest or on mild exertion, limited exercise capacity and signs of organ hypoperfusion (e.g., increased lactate levels, altered mental status, oliguria, chronic hypotension).^26^, ^27^ These differences account for more severe cardiac dysfunction, including RV dysfunction and signs of right HF (RHF) affecting most AdvHF patients. Refractory atrial and sustained ventricular arrhythmias may also trigger clinical exacerbation and often do not respond to anti‐arrhythmic drugs.^28^ This status requires urgent ablation or cardioversion–defibrillation (DC) shocks, possibly resulting in haemodynamic deterioration.^6^, ^7^, ^29^ Hospitalization rates cannot be used to distinguish the two phenotypes. Instead, the causes of hospitalization may be considered discriminating factors: WHF episodes include precipitating factors not necessarily associated with haemodynamic deterioration, while AdvHF hospitalizations are related to haemodynamic deterioration^25^, ^28^, ^29^ (*Figure* *2*).

### Haemodynamic profile

In WHF, haemodynamic alterations are primarily characterized by moderate and transient indices of increased LV filling pressure (e.g., elevated E/e' ratio) and mild, reversible pulmonary hypertension. Importantly, cardiac output is typically preserved with minimal or no signs of a low output state. With adequate treatment, haemodynamic values and congestion can be significantly improved or even normalized.^30^, ^31^, ^32^, ^33^

In AdvHF, the haemodynamic profile is far more severe and persistent. Patients exhibit persistently elevated LV filling pressures and severely reduced cardiac output and stroke volume. This leads to progressive RV dysfunction, severe tricuspid regurgitation and pre‐ and post‐capillary pulmonary hypertension. Invasive haemodynamic monitoring via a pulmonary artery catheter is often useful to guide the use of inotropes, vasopressors or MCS and is a cornerstone for assessing candidacy for HTx or LV assist device (LVAD) implantation^34^, ^35^, ^36^, ^37^, ^38^ (*Table* *2*).

**Table 2:** Specific clinical haemodynamic, arrhythmic burden in advanced versus worsening HF is associated with distinct clinical presentation, haemodynamic conditions, arrhythmic risk, and diagnostic and management features.

Category	Worsening heart failure	Advanced heart failure
Clinical presentation	‐ No hypoperfusion ‐ Resting dyspnoea ‐ Reduced exercise tolerance ‐ Preserved neurological status	‐ Signs of hypoperfusion (cold skin, low BP) ‐ Severe dyspnoea ‐ Stable but severely limited physical tolerance ‐ Mental confusion
Congestion signs	‐ Moderate signs of increased CVP ‐ Pulmonary rales regress with decongestive therapy	‐ Jugular vein distension ‐ Marked limb oedema ‐ Hepatojugular reflex ‐ Hepatomegaly ‐ Persistent pulmonary rales ‐ Pleural and abdominal effusion
Haemodynamic profile	‐ Reduced or preserved CO ‐ Increased LV filling pressure ‐ Transient pulmonary pressure rise ‐ Reversible CVP elevation	‐ Reduced CO ‐ Persistent increase in wedge and pulmonary pressures ‐ Elevated CVP ‐ Increased pulmonary and systemic resistance
Arrhythmic profile	‐ Moderate risk of arrhythmias ‐ VT events responsive to DC shock ‐ Possibility for ablation or device therapy	‐ High risk of VT/VF despite ICD ‐ Recurrent VT poorly responsive to DC shock
Renal condition	‐ Transient WRF ‐ Related to congestion and therapy ‐ Good diuretic response	‐ Common CKD ‐ Tubular resistance ‐ Poor response to diuretics ‐ Requires multiple nephron blockades
Ultrasound features	‐ Milder systolic/diastolic dysfunction ‐ No RV dysfunction ‐ No permanent VCI distension ‐ Transient B‐lines ‐ Reversible renal venous flow changes	‐ Severe systolic and diastolic dysfunction ‐ RV dysfunction ‐ Significant tricuspid regurgitation ‐ VCI distension and reduced collapse ‐ Persistent pulmonary congestion ‐ Altered renal venous flow
Hospital monitoring	‐ Non‐invasive (mainly ultrasound) ‐ Occasional use of central vein access	‐ Invasive monitoring with central venous access ‐ Continuous measurement of wedge, pulmonary and venous pressures
Laboratory markers	‐ Transient rise in NP and adrenomedullin ‐ Normal liver function and albumin ‐ Moderate renal marker elevation ‐ Possible iron metabolism disturbance	‐ Persistent elevation of NP, pro‐adrenomedullin, ST2, CA‐125, GDF‐15, interleukins ‐ Liver, kidney, tubular markers altered ‐ Electrolyte disorders ‐ Low albumin ‐ Disrupted iron metabolism
Treatment	‐ Tolerates RAASi, ARNI, beta‐blockers, MRA ‐ Possible addition of vericiguat ‐ IV diuretics only during acute episodes ‐ No need for inotropes ‐ Suitable for device implantation and planned follow‐up ‐ Venous access only during hospitalization	‐ Poor tolerance to RAASi, ARNI, beta‐blockers, MRA ‐ Requires high‐dose loop diuretics (IV cycles) ‐ Often needs inotropes (e.g., levosimendan) ‐ Poor response to CRT and ICD ‐ Consider ECMO, ultrafiltration, LVAD or HTx ‐ Central vein access often needed long term

Abbreviations: ARNI, angiotensin receptor–neprilysin inhibitor; BP, blood pressure; CA‐125, cancer antigen 125; CKD, chronic kidney disease; CO, cardiac output; CRT, cardiac resynchronization therapy; CVP, central venous pressure; DC, cardioversion–defibrillation; ECMO, extracorporeal membrane oxygenation; ICD, intracardiac defibrillator; GDF‐15, growth differentiation factor 15; HTx, heart transplant; IV, intravenous; LV, left ventricle; LVAD, left ventricular assist device; MRA, mineralocorticoid receptor antagonist; NP, natriuretic peptide; RAASi, renin–angiotensin aldosterone system inhibitors; RV, right ventricle; ST2, soluble suppression of tumorigenicity 2; VCI, inferior cava vein; VF, ventricular fibrillation; VT, ventricular tachycardia; WRF, worsening of renal function.

### Laboratory and circulating biomarkers

**Figure 3:**
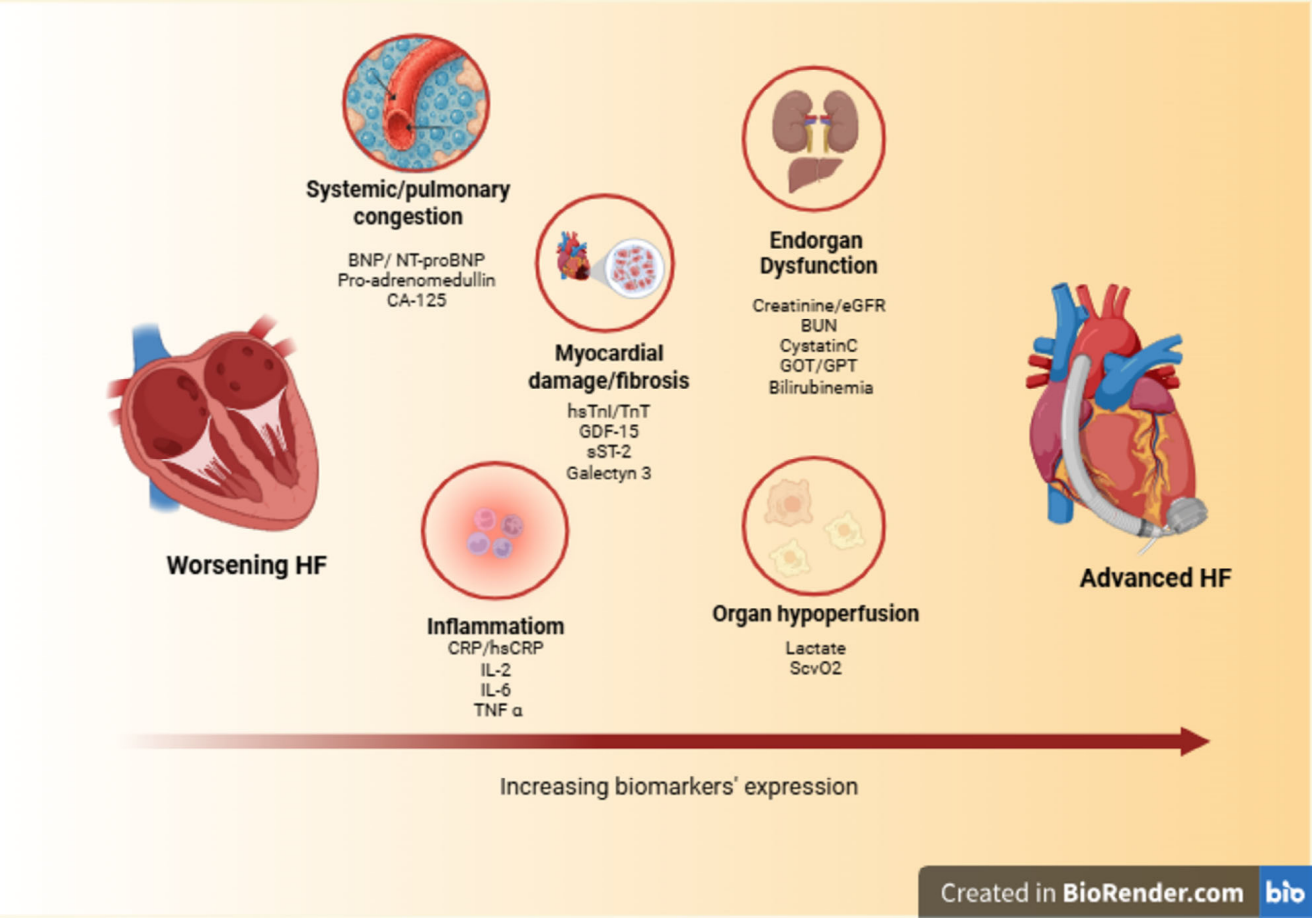
Different diagnostic and laboratory marker profiles in WHF versus AdvHF. *Abbreviations*: BNP, brain natriuretic peptide; BUN, blood urea nitrogen; CA‐125, cancer antigen 125; CRP, C‐reactive protein; eGFR, estimated glomerular filtration rate; GDF‐15, growth differentiation factor 15; GOT, glutamic‐oxaloacetic transaminase; GPT, glutamic‐pyruvate transaminase; IL, interleukin; NT‐proBNP, N‐terminal pro‐B‐type natriuretic peptide; sST2, soluble suppression of tumorigenicity‐2; TNFα, tumour necrosis factor α; TnI, troponin I; TnT: troponin T.

Both WHF and AdvHF share a laboratory marker profile indicative of severe cardiac impairment, reflected by elevated natriuretic peptides due to increased ventricular wall stress and congestion.^39^ A chronic pro‐inflammatory state is common in HF, evidenced by elevated circulating biomarkers such as C‐reactive protein, interleukin‐1 and interleukin‐6.^40^, ^41^ In WHF, these markers may also signal underlying infectious triggers. Markers of congestion and cardiac damage, including pro‐adrenomedullin, cancer antigen 125 and galectin‐3, tend to be more elevated in AdvHF than in WHF, although definitive thresholds have yet to be established.^42^ Additionally, systemic organ hypoperfusion and end‐organ dysfunction—manifested by elevated lactate levels, kidney and liver function abnormalities, hypoalbuminaemia and electrolyte imbalances—are typically observed in AdvHF. These markers are crucial for guiding therapeutic decisions and risk stratification. Finally, markers of apoptosis, myocardial injury and altered metabolic function, such as growth differentiation factor‐15 (GDF‐15), troponin I and suppression of tumorigenicity 2, are frequently elevated in both conditions (*Figure* *3*).^43^ Elevated circulating GDF‐15 levels were also associated with a higher prevalence of cachectic status, RV dysfunction, congestion and increased risk in AdvHF regardless of natriuretic peptide values.

## Therapeutic strategies

### Acute treatment

Patients presenting with WHF often have suboptimal or underdosed neurohormonal antagonist therapy, making urgent visits or hospitalizations an opportunity to initiate new medications or optimize existing treatments, particularly diuretics.^44^, ^45^ Among patients requiring HFH, a significant increase in IV diuretic dosing—without necessarily implementing a multi‐nephron blockade strategy—may be sufficient to decongest the patient and achieve clinical euvolaemia. For those requiring only urgent ambulatory visits, where IV diuretic therapy may not be readily available, doubling the oral diuretic dose is often an effective approach.^17^, ^46^ In these subjects, the recurrent episode could be essential to identify and mitigate triggering causes, to promote stability and avert future hospitalizations. Additional pharmacological or interventional procedures may be recommended after acute episode resolution.^16^, ^45^

In AdvHF, diuretics should be administered intravenously, often in combination with a thiazide or IV acetazolamide to enhance diuretic efficiency.^5^, ^27^, ^47^ In cases of diuretic resistance, ultrafiltration may be necessary to alleviate interstitial fluid overload. Patients with AdvHF frequently require inotropic or vasopressor support (e.g., noradrenaline, dobutamine, levosimendan) to maintain organ perfusion and sustain a mean systolic blood pressure >65 mmHg. Serial haemodynamic assessments to measure pulmonary capillary wedge pressure (PCWP), right atrial pressure (RAP) and cardiac output monitoring are essential for optimizing management.^35^, ^48^, ^49^ For patients in advanced stages, inotropes can serve as a bridge to heart HTx or LVAD implantation or be used for palliative care. MCS options such as extracorporeal membrane oxygenation (ECMO) or intra‐aortic balloon pump may be employed to improve stroke volume, reduce cardiac workload and optimize vascular resistance as a bridge‐to‐decision strategy (e.g., transition to durable LVAD or HTx).^50^, ^51^ By mitigating end‐organ dysfunction, temporary MCS can provide critical time to determine candidacy for definitive therapy. However, the optimal timing for transitioning from temporary MCS to durable LVAD or HTx remains an area of ongoing research.^52^, ^53^ Given the limited availability of donor hearts and the growing population of patients with AdvHF, durable LVADs have become increasingly utilized as either bridge‐to‐transplant or destination therapy. In the United States, the centrifugal‐flow HeartMate 3 is the most commonly implanted LVAD for both indications.^54^ Despite advancements in LVAD technology, challenges remain, including risks of stroke, bleeding, driveline infections and RV failure, with survival rates of 84%, 78% and 58% at 1, 2, and 5 years, respectively.^28^, ^55^ Nevertheless, the exact AdvHF prevalence around the world is not yet established due to the lack of solid epidemiological data, different criterion definitions and few population‐based studies; the prevalence using ESC criteria is calculated at about 5.1%–7.7% per year.^56^ In this framework, despite statistics suggesting that around 500,000 individuals—‘may need’ LVAD implantation, only 0.03–0.05 receive HTx or LVAD in the United States.^57^ Less common bridge‐to‐transplant options include the extracardiac ultrafiltration and ECMO mitral transcatheter edge‐to‐edge repair (MTEER) with total artificial heart, typically reserved for patients with severe biventricular dysfunction.^58^, ^59^

### Chronic treatment

In routine clinical practice, patients with WHF are typically not optimally managed with GDMT during stable periods and present with a less compromised haemodynamic status, facilitating the up‐titration of the four pillars currently utilized for the long‐term treatment of HFrEF.^60^, ^61^, ^62^ In these patients, it is also crucial to identify and address precipitating factors to facilitate stabilization and to prevent future HFH. The acute episode may also provide an opportunity to introduce additional therapies, both pharmacological (e.g., vericiguat and/or ivabradine)^63^ and interventional (e.g., percutaneous coronary revascularization, cardiac resynchronization therapy implantation, mitral transcatheter edge‐to‐edge repair [MTEER] and tricuspid transcatheter valve interventions).^59^, ^60^, ^64^ Growing evidence also suggests that IV iron therapy in patients with iron deficiency reduces the risk of rehospitalization.^65^

Conversely, patients with AdvHF are more often already on optimal medical therapy, despite many of them being unable to tolerate GDMT due to impaired clinical and haemodynamic profiles.^66^ Severe impairment of cardiac pump function can lead to chronically low blood pressure levels, making the administration, tolerance and target dose achievement of GDMT challenging.^5^, ^67^ The simultaneous administration of the four pillars utilized in patients with low cardiac output or severely reduced glomerular filtration may not be tolerated.^68^ Similarly, AdvHF patients are often unable to tolerate one or more multiple foundational GDMTs, which are frequently down‐titrated or ineffective.^6^, ^69^ Multiple mechanisms, such as organ hypoperfusion, neurohormonal activation, excessive tubular sodium reabsorption, chronic inflammation, and structural alterations, can contribute to kidney dysfunction and diuretic resistance, making the treatment of congestion difficult.^5^, ^6^, ^50^, ^69^, ^70^ A comprehensive approach to the patient includes evaluation for long‐term MCS (e.g., LVAD) or HTx candidacy by an AdvHF centre or symptom palliation in elderly patients or those with concomitant systemic diseases with limited life expectancy.^28^, ^53^, ^71^, ^72^ Of note, a subset of patients with durable MCS experiences significant myocardial recovery (‘bridge to recovery’), sometimes allowing for device explant.^54^, ^55^

Therefore, AdvHF represents a state of decline that is often but not universally irreversible as mechanical unloading can alter this course in select individuals. In this setting, recurrent and planned inotropic therapy infusion should be attempted in order to partially restore haemodynamic condition in patients awaiting HTx or LVAD, despite the treatment being affected by relevant arrhythmic complications and hospitalization.^73^

In patients with reduced life expectancy, palliative care interventions should be considered to reduce hospitalizations, despite no significant effect on mortality and quality of life having been demonstrated^74^. Current management should be applied in patients with end‐stage HF with a prognostic estimation of <6 months, and it consists of patient's symptom alleviation, psychological support, management of comorbidities and tolerance of frailty status^75^ (*Table* *3*).

**Table 3:** Therapeutic options, opportunities and limitations in worsening versus advanced heart failure.

Therapy	WHF	AdvHF
RAASi, ARNI and β‐blockers	Initiation and titration opportunity	Already inserted but incompletely tolerated and low‐dose amount
Loop diuretics	Temporarily increased amount but no high dose in stable period	High‐dosage administration with need of repetitive intravenous cycle
SGLT2i	May be added and should not be interrupted during the acute phase	No data in AdvHF patients, no significant adverse effects
MRA	Good tolerance and opportunity for initiation	Potentially dangerous in patients with severe CKD
Vericiguat	Opportunity for therapy addition	Potentially dangerous in low‐BP profiles or with hypotension
Levosimendan	May be administered but not usually needed	Needs repetitive treatment
Anti‐arrhythmic drug	Not usually administered or given in acute period	Class III drugs already taken for ventricular arrhythmia prevention
ICD/CRT	Usually not implanted yet; may be evaluated for implantation	Already implanted because of high arrhythmic risk or severe dysfunction
Mitraclip or TAVI	Evaluation for treatment and symptom recovery	Previously inserted or last chance for QoL improvement
TTVR	Not usually indicated	Possibly inserted for symptom alleviation and QoL amelioration
Palliative care	Not advised	Advised in end‐stage HF and relevant frailty status

Abbreviations: ARNI, angiotensin receptor–neprilysin inhibitor; CRT, cardiac resynchronization therapy; ICD, intracardiac defibrillator; MRA, mineralocorticoid receptor antagonist; RAASi, renin–angiotensin aldosterone system inhibitors; QoL, quality of life; SGLT2i, sodium glucose transporter inhibitor; TAVI, transcatheter aortic valve implantation; TTVR, transcatheter tricuspid valve replacement.

## Addressing knowledge gaps and defining future directions

Despite considerable progress in understanding and managing WHF and AdvHF, several critical knowledge gaps remain. One of the most pressing challenges is the absence of a universally accepted classification system that differentiates WHF from AdvHF. Currently, there is no standardized diagnostic framework that distinguishes these two entities in clinical practice. The reliance on subjective clinical criteria, rather than objective haemodynamic parameters, complicates the early identification and appropriate management of these conditions. Future research should focus on refining risk stratification tools that integrate biomarkers, advanced imaging techniques and haemodynamic assessments to provide a more precise characterization of these conditions.^3^, ^15^, ^18^

Another significant gap lies in understanding the pathophysiological mechanisms driving the transition from WHF to AdvHF. While neurohormonal activation, myocardial remodelling and end‐organ dysfunction are known to contribute to HF progression, the molecular and genetic mechanisms underlying this transition remain poorly understood.^76^, ^77^ Advances in omics technologies, including genomics, proteomics and metabolomics, could uncover novel biomarkers and therapeutic targets that might help prevent the progression from WHF to AdvHF.

The role of emerging pharmacological and interventional therapies in the management of WHF and AdvHF is another area of active investigation. Many patients with AdvHF fail to tolerate optimal doses of medications due to haemodynamic instability. The development of novel agents, such as myosin activators, soluble guanylate cyclase stimulators and neprilysin inhibitors, holds promise for improving outcomes. However, their efficacy and safety in these high‐risk populations require validation through large‐scale, randomized clinical trials.^78^ Future studies should focus on creating individualized treatment algorithms that optimize therapy selection and timing to improve both survival and quality of life. Finally, there is an impelling need for a more comprehensive and arbitrary algorithm to discern the two phenotypes by the application of strict criteria accounting for patients' history, cardiac dysfunction severity, diagnostic threshold and causes of clinical destabilization (Table S1). The emerging use of artificial intelligence and machine learning presents a significant opportunity to enhance risk prediction and management. By analysing large datasets from electronic health records, wearable devices and imaging studies, artificial intelliegence (AI)‐driven approaches could facilitate the early detection of these conditions and enable more personalized, data‐driven management strategies.^79^

## Conclusions

AdvHF and WHF represent distinct trajectories in the natural history of HF, each with related yet distinct clinical and pathophysiological characteristics. While significant progress has been made in improving care for these patients, specific longitudinal studies understanding distinguished management strategies, disease triggers, and outcomes are still lacking. Challenges warrant detailed investigations concerning the lack of a universally accepted classification system, incomplete understanding of precipitating mechanisms, and limitations in current treatment strategies. Future research should focus on refining risk stratification tools, incorporating biomarkers and advanced imaging modalities, and leveraging emerging technologies, such as AI and remote monitoring, to enable early detection and proper management of these distinct clinical entities.

## Supporting information


**Table S1:** A roadmap for future investigations and unresolved questions.
