# Supplementary material for: Worsening versus advanced heart failure: Management and challenges
Source: ESC Heart Fail. 2025 Oct 4;12(6):3856–68. doi: 10.1002/ehf2.15437 (PMC12719834; doi:10.1002/ehf2.15437)
Supplement: Supplementary file 1 — Table S1: A roadmap for future investigations and unresolved questions. [file EHF2-12-3856-s001.docx]

**Supplementary Table 1:**

| **Category** | **Worsening heart failure** | **Advanced heart failure** |
| --- | --- | --- |
| **How to assess the cause of destabilization** | Include cardiac congestion and extracardiac disorders capable of induce clinical deterioration | Consider only hemodynamic deterioration and recurrent congestion related to proper cardiac conditions |
| **How to define by cardiac function and reserve** | Unchanged cardiac condition across recurrence episodes or include patients with significant functional deterioration with uncomplete recover | Unreversible cardiac dysfunction with complete lost of cardiac reserve, biventricular dysfunction or include patients with partial improvement after inotropic lusitropic therapy cycle |
| **How to define deterioration episodes** | Consider both urgent visit ED access and hospitalization not necessarly requiring IV diuretic or include only patients need IV escalation diuretic dose | Refer patients requiring hospitalization for cardiac deterioration or extend to patients with planned repetitive ambulatory infusion treatment |
| **How to discern by appropriate diagnostic strategy** | Definition by biomarkers . echocardiographic ultrasound index of congestion and invasive measurement thresholds | Recognized INTERMACS definition but avoid overlap with some WHF features |
| **How to define by tailored treatment** | Permanent or transient diuretic escalation dose, introduction of new drug (vericiguat omecamtiv mecarbil ivabradine) or titration of traditional therapy. CRT or ICD implantation may be considered | Universal recognition of traditional treatment intolerance, repetitive ambulatory infusion therapy, list for HTX / LVAD or palliative care; consider mitraclip or tricuspid transcatheter valve replacement for those with relevant secondary valve defects |
